# Profound Neuropathy after Penetrating Transection of the Sciatic Nerve by Femoral Cerclage Wire: Illustrative Case and Management Strategy

**DOI:** 10.1089/neur.2024.0156

**Published:** 2025-01-30

**Authors:** John K. Yue, Jun Yeop Oh, Alexander A. Aabedi, Jia-Shu Chen, Kenneth X. Probst, Vinil N. Shah, Rosanna L. Wustrack, Line G. Jacques

**Affiliations:** ^1^ Department of Neurological Surgery, University of California, San Francisco, San Francisco, California, USA.; ^2^ Department of Radiology and Biomedical Imaging, University of California, San Francisco, San Francisco, California, USA.; ^3^ Department of Orthopedic Surgery, University of California, San Francisco, San Francisco, California, USA.

**Keywords:** clinical decision-making, intraoperative neurophysiological monitoring, neuroimaging, sciatic neuropathy, surgical diagnostic technique, total hip arthroplasty

## Abstract

Sciatic nerve injury associated with total hip arthroplasty (THA) confers chronic and progressive disability. Mechanisms of injury are heterogeneous and management nuances are often case-specific. We discuss a Sunderland Type 4 sciatic nerve transection by femoral cerclage wire from prior THA to highlight optimal clinical strategies when approaching complex cases. A 65-year-old woman presented to the neurosurgery clinic with worsening, medically refractory right sciatic sensorimotor neuropathy that began 1 year after ipsilateral hip arthroplasty. Neurological examination detected weakness in ankle dorsiflexion/plantarflexion and foot inversion/eversion (motor scale 2–3/5), toe extension/flexion (1/5), foot numbness, and hyperesthesia. Electromyogram confirmed sciatic neuropathy. Magnetic resonance neurogram (MRN) showed a thickened right sciatic nerve abutting a femoral cerclage wire, which appeared contiguous on reconstructed computed tomography imaging. Intraoperatively, the wire was discovered to have clearly transected and remained lodged within the sciatic nerve, requiring orthopedic surgery consultation and wire cutdown at the transection site. The surrounding neuroma was excised and the defect was reconstructed using nerve allograft interposition. Intraoperative neurophysiology monitoring (IONM) signals remained stable. Radiographs confirmed uncomplicated wire disconnection. The patient was discharged home the next day and reported significant symptomatic relief at 1-month follow-up. Delayed presentation of sciatic nerve transection by femoral cerclage wire with ongoing neural compression is rare. The anatomy of injury can be high risk, impelling thoughtful operative planning in THA as well as neuroplasty cases. Strategies include preoperative MRN to evaluate the pathoanatomy of nerve injury, neurosurgery and orthopedic surgery comanagement, and multimodal IONM to reduce risks of intraoperative neural injury and optimize outcomes.

## Introduction

Sciatic neuropathy after total hip arthroplasty (THA) surgery confers significant functional disability, most commonly motor foot weakness impairing ambulation. Etiologies of associated iatrogenic sciatic nerve injuries including limb lengthening or displacement procedures, compression from subfascial hematoma, and thermal injuries from cementation are likely underreported.^1,2^ Less than 50% of cases experience full functional recovery even when promptly diagnosed.^3,4^ This is especially true after nerve transection.^5^ With demand for THA increasing annually,^6,7^ improved pathoanatomical understanding of iatrogenic sciatic nerve injuries is crucial for devising management approaches. Circumferential femoral cerclage wires, commonly used to enhance the stability, alignment, and fixation of proximal femoral bone fractures, or as prophylactic banding in non-cemented THA, are mechanically effective but not without risk of damaging neurovascular and soft tissue structures within its proximity. While vascular injuries are secondary to cerclage wires, these have been estimated at 2–7%,^8,9^ characteristics of associated iatrogenic nerve injuries are seldom described, with only two published reports to date.^10^

Herein, we present an unusual case of a patient with progressive right leg and foot weakness, numbness, and pain secondary to an oblique, transverse traumatic sciatic nerve transection with ongoing nerve compression and traction by femoral cerclage wire placed during THA 1 year ago. To our knowledge, this is the first report of a sciatic nerve transection by cerclage wire and highlights the operative considerations and strategies for this unusual injury.

## Case Report

### History and physical examination

A 65-year-old woman with rheumatoid arthritis was referred to our institution for evaluation of chronic, worsening right sciatic sensorimotor neuropathy 1 year after right THA at another institution. She had full strength in bilateral lower extremities before THA and noticed new right foot weakness, paresthesias, and pain postoperatively, which were progressive and refractory to physical therapy and multimodal pain medications.

Physical examination revealed right calf muscle atrophy and low muscle tone in the calf and foot. As per the Medical Research Council (MRC) motor scale (M; range 0–5), the neurological exam was notable for deficits in knee flexion M4, ankle dorsiflexion M2, ankle plantarflexion M3, foot inversion M2, foot eversion M3, toe extension M1, and toe flexion M1. Sensory examination demonstrated numbness to light touch along the right calf and the dorsal and plantar surface of the entire right foot with associated paresthesia and hyperesthesia, corresponding to MRC sensory scale (S; range 0–4) S2+. Her left lower extremity had no deficits.

### Diagnostic approach

**FIG. 1.**
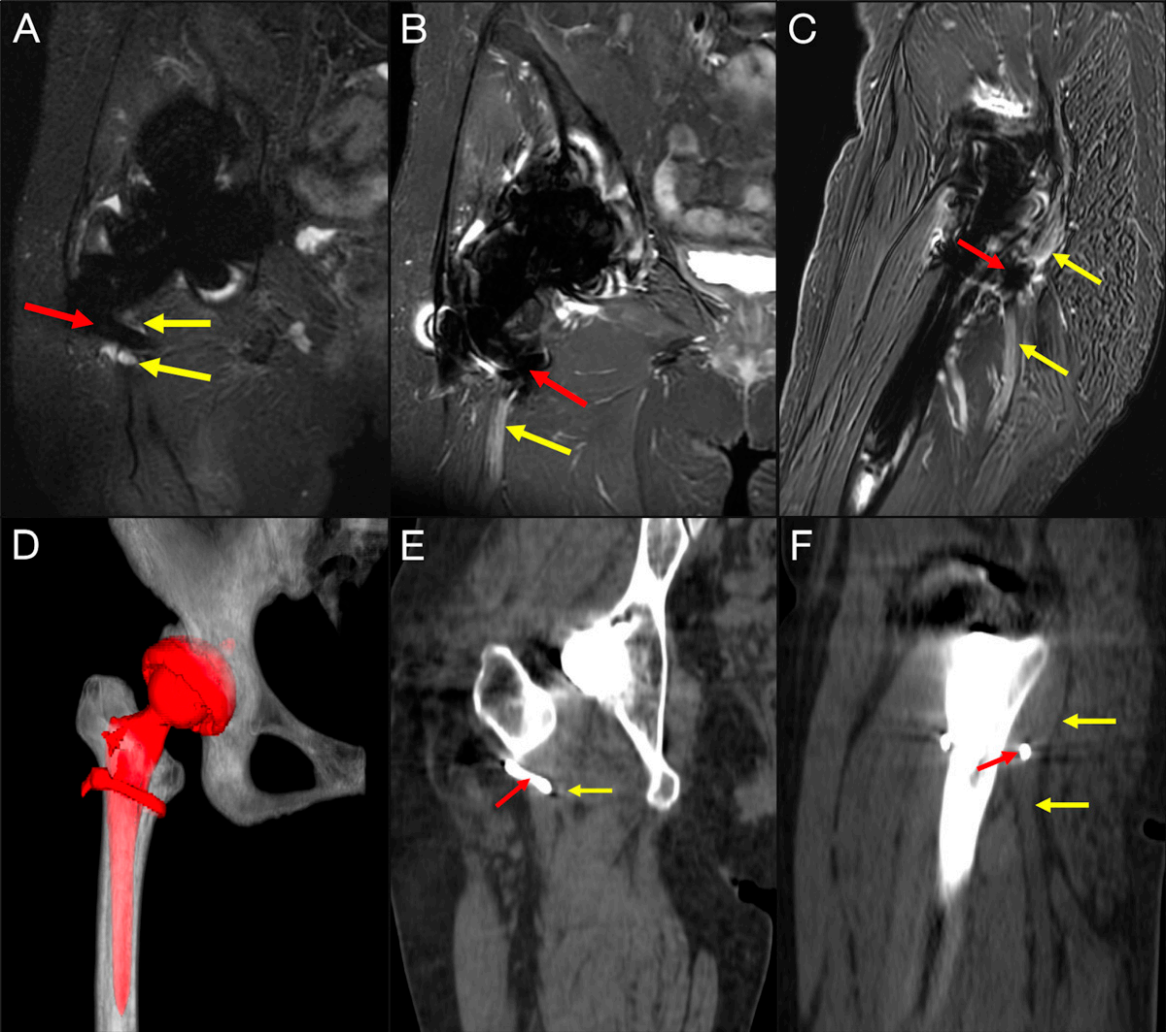
Preoperative magnetic resonance neurogram and computed tomography. Preoperative magnetic resonance neurogram showed abnormal features in the right sciatic nerve at the hip level. There is a discontinuity and abnormal thickening of the sciatic nerve (yellow arrow) with increased T2-weighted signal intensity adjacent and distal to the site of femoral cerclage wire placement (red arrow; metal artifact). (**A** and **B**) Coronal T2, left side of image = right side of patient; (**C**) sagittal T2, left side of image = anterior to patient. Preoperative computed tomography (CT) scan and three-dimensional reconstruction of the right lower extremity showed significant thickening of the sciatic nerve (yellow arrow) with the posterior aspect of the cerclage wire (red arrow) contiguous with the sciatic nerve. (**D**) Three-dimensional reconstruction of the right total hip arthroplasty implant and femoral cerclage wire in red color; (**E**) coronal CT, left side of image = right side of patient; and (**F**) sagittal CT, left side of image = anterior to patient.

Magnetic resonance neurogram (MRN) with and without contrast of the pelvis showed abnormal thickening, increased T2 signal, and subtle enhancement of the right sciatic nerve adjacent and distal to the femoral cerclage wire (Fig. 1A–C). Noncontrast computed tomography (CT) and 3-dimensional reconstruction of the pelvis and right lower extremity revealed that the posterior aspect of the wire was contiguous with the markedly thickened sciatic nerve (Fig. 1D–F). Right lower extremity electromyogram (EMG) showed a moderate density of fibrillation potentials in the biceps femoris, tibialis anterior, gastrocnemius, and semitendinosus muscles, confirming sciatic nerve injury with signs of reinnervation in the right biceps femoris (long duration motor unit action potentials [MUAPs] and nascent MUAPs), and ongoing degeneration (reduced MUAPs) in the tibialis anterior and gastrocnemius.

### Preoperative discussion and informed consent

Risks, benefits, and alternatives of surgical decompression, neuroma excision, and nerve repair of the right sciatic nerve were reviewed with the patient, including possible disconnection and/or removal of the cerclage wire to achieve neural decompression. The patient was counseled that reinnervation procedures 12 months after axonotmesis may provide limited sensorimotor benefit, given the split injury to the distal sciatic nerve branches and the severity of muscle degeneration detected by EMG and the MRC motor scale. The patient understood these limitations and decided to proceed with nerve decompression and repair with the goal of reducing neuropathic pain. Efficacy and outcomes of autograft/allograft repair were discussed. While evidence favoring autograft reconstruction of complex peripheral nerve injuries and injuries ≥3 cm in length is recognized,^11^ the patient requested to undergo allograft reconstruction rather than an autograft donor site. The patient provided informed consent for surgery and medical research, including publication.

### Operative techniques and findings

**FIG. 2.**
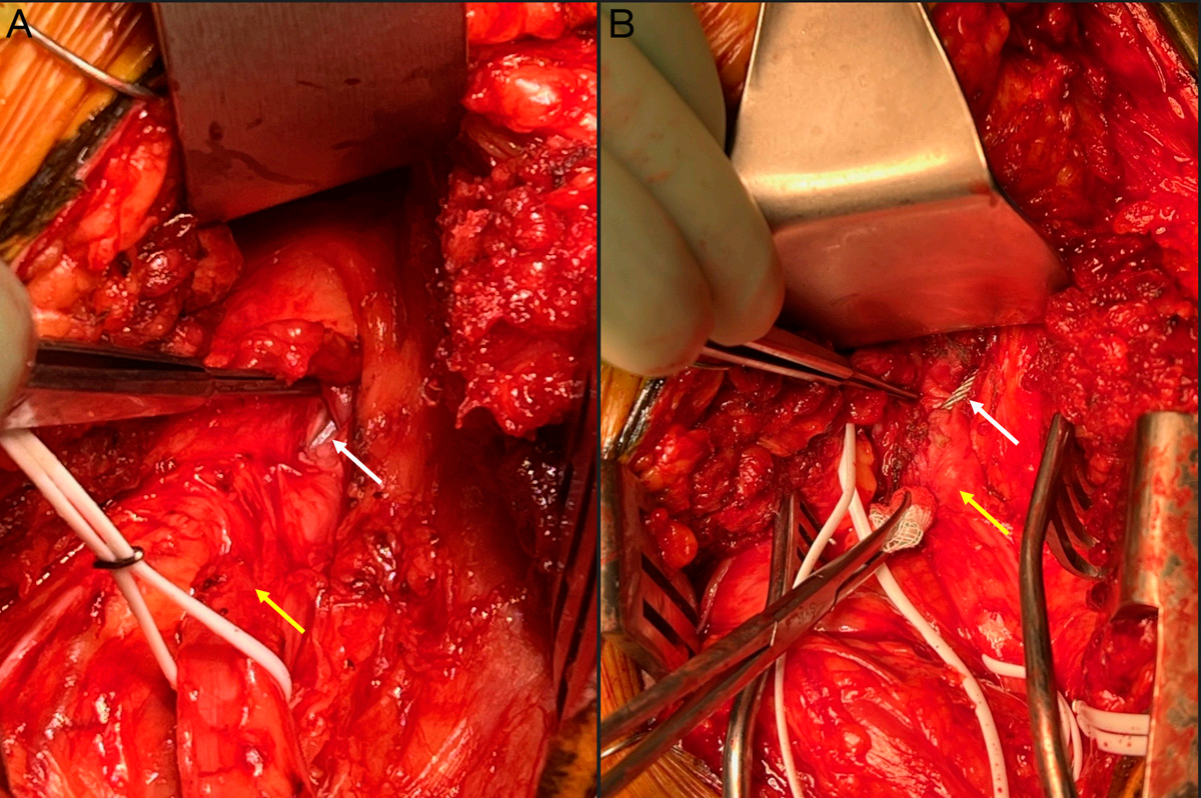
Intraoperative finding of cerclage wire transecting sciatic nerve. Intraoperative photographs showed traumatic transection of the sciatic nerve (yellow arrow) by the femoral cerclage wire (white arrow), which transected the nerve in a superolateral to inferomedial trajectory. (**A**) Earlier stage of exposure and (**B**) later stage of exposure; right side of image = lateral aspect of the right buttock and upper thigh. Two-thirds of the nerve diameter was severed, leaving one-third intact in the posterior tibial and peroneal divisions. The wire was surgically disconnected using a wire cutter lateral to its entry site into the sciatic nerve.

**FIG. 3.**
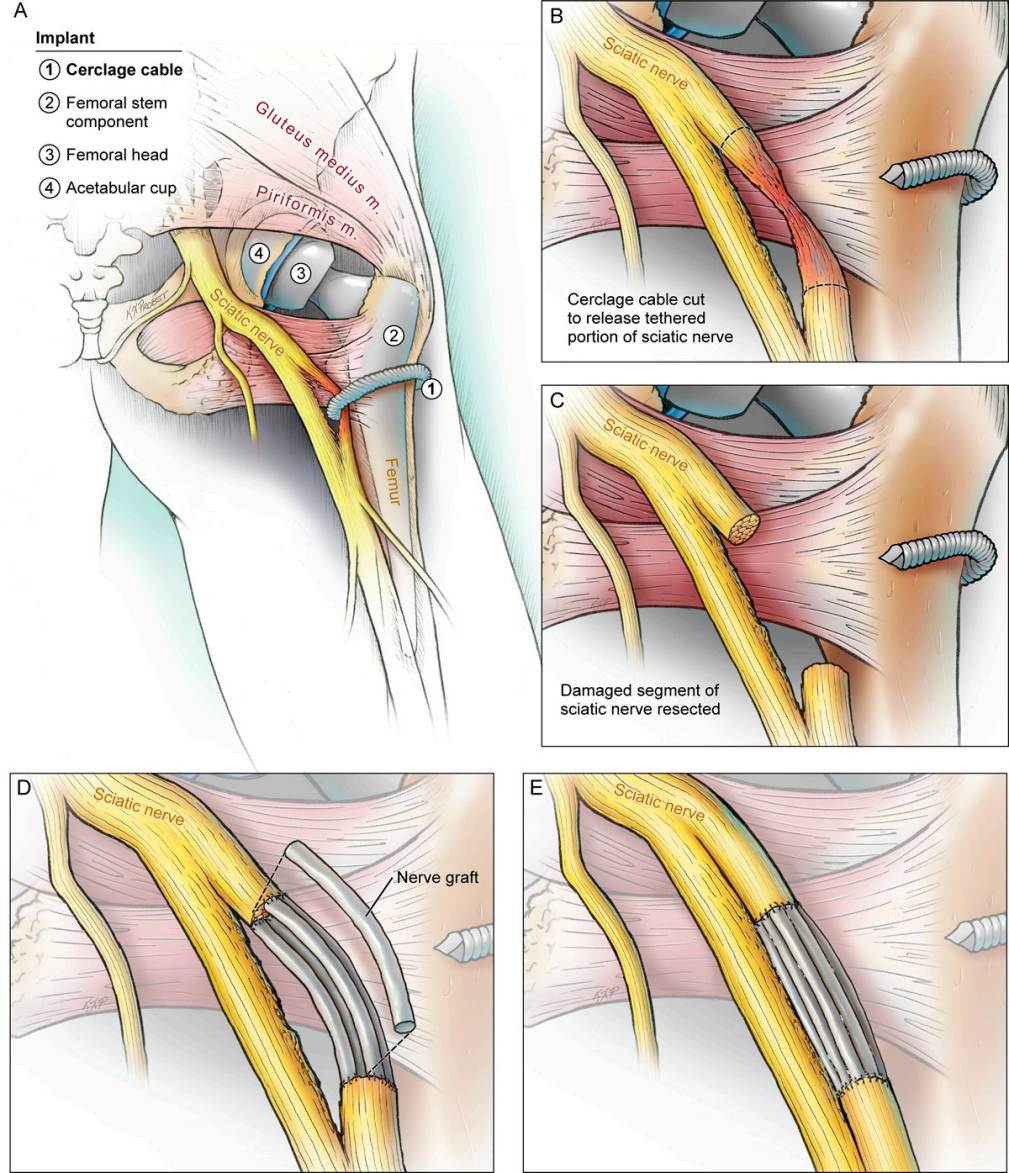
Illustrative anatomical schematic of sciatic nerve injury and repair. Illustration demonstrating the anatomical relationship of the sciatic nerve, femoral cerclage wire, nature of transection and compression, and subsequent nerve repair with allograft. (**A**) Global view of patient’s right lower extremity with femoral implant and cerclage wire transecting the sciatic nerve transversely across approximately two-thirds of the nerve’s cross-sectional diameter in both posterior tibial and peroneal divisions, causing a Sunderland Type 4 axonotmesis. (**B**) Visualization of the injured sciatic nerve fascicles following cutting of the cerclage wire. (**C**) Visualization of the sciatic nerve following neurolysis and trimming of the traumatic neuroma to facilitate graft repair. (**D**) Visualization of the sciatic nerve during allograft repair with six 4–5 mm diameter × 30 mm length human nerve allograft via interposition using 8-0 prolene sutures. (**E**) Visualization of the sciatic nerve end product following graft repair.

The patient was positioned prone under general anesthesia, and the incision was made along the previous THA incision from the right lower buttock to the posterior upper thigh. The sciatic nerve was carefully exposed proximal and distal to the expected site of injury identified on preoperative imaging at the subtrochanteric femur at the buttock level, and vessel loops were placed around the nerve. The cerclage wire was identified coursing superolaterally to inferomedially across the posterior subtrochanteric area, with transection through the sciatic nerve in an oblique transverse direction, causing a Sunderland Type 4 axonotmesis. The metal wire had severed approximately two-thirds of the nerve’s cross-sectional diameter in both the posterior tibial and peroneal divisions (Fig. 2A, B), leaving no possibility to free the nerve without cutting the wire (Fig. 3A).

The orthopedic surgery service was consulted intraoperatively for evaluation of cerclage wire disconnection. Since the patient was 1-year post-THA without fractures or hardware failure on preoperative CT, and there were no alternatives to relieve the nerve from injury, the cerclage wire was carefully disconnected using a surgical wire cutter at the site of sciatic nerve transection, which then freed the nerve. As the indication for neural decompression was achieved, and circumferential visibility and access were not possible, we did not further pursue removal of the cerclage wire.

The visible course of the sciatic nerve was mapped using intraoperative neurophysiology monitoring (IONM). There was a rim of traumatic neuroma surrounding the transection site, which was carefully and sharply trimmed until normal fascicular structures were clearly observed at the proximal and distal ends of the partial transection site (Fig. 3B, C). The traumatic neuroma was sent to pathology with a confirmatory diagnosis. Following internal neurolysis, six 4–5 mm diameter × 30 mm length human nerve allografts (Axogen Corporation, Alachua, Florida, USA) were interposed across the defect of the injured nerve segment using 8-0 prolene sutures (Fig. 3D, E). The uninjured segment of the nerve was preserved. The surgical site was copiously irrigated with antibiotic crystalloid solution followed by standard multilayered closure. Motor-evoked potentials, somatosensory-evoked potentials, and EMG signals remained unchanged throughout the case.

The postoperative course was uncomplicated. The hip radiograph showed the disconnected femoral cerclage wire. The patient noted improved neuropathic pain compared to presurgery and was discharged home the next day. At 1-month clinic follow-up, the patient had a stable motor neurological exam and reported further improvement in her sciatic nerve distribution pain and paresthesias.

## Discussion

### Case overview

We present the rare case of direct sciatic nerve transection from previous femoral cerclage wire during THA causing chronic foot weakness, sensory deficits, and neuropathic pain for 1 year prior to surgical neuroplasty at our institution. Cerclage wires, employed in THA to secure the femoral component and enhance the stability of the prosthesis, can inadvertently compromise critical neurovascular structures. Cases of sciatic neuropathy due to entrapment by cerclage wire at the lesser trochanter have been reported^2^; however, these did not involve direct penetration or rupture of the nerve. Importantly, our case illustrates another mechanism through which cerclage wires can pose rare but serious risks to peripheral nerves of the hip and lower extremities, emphasizing the need for surgical vigilance during THA surgery and when evaluating undifferentiated traumatic nerve injuries. After evaluating bone and hardware integrity on preoperative CT and magnetic resonance imaging (MRI), our decision-making process to disconnect the cerclage wire at the sciatic nerve transection site highlights the importance of coordination between neurosurgeons and orthopedic surgeons to prevent further nerve injury or bony implant failure.

### Nerve injuries associated with THA and cerclage wiring

Nerve injuries associated with THA range from 1% to 4%, and the sciatic nerve accounts for 90% of them.^3^ The sciatic nerve may be particularly susceptible due to its proximity to bony implants during the posterior or posterolateral approach, its steep inferior projection through the greater sciatic notch, and the limited soft tissue space along its course between the ischium and the lesser trochanter.^12,13^ Several factors are implicated in THA-associated sciatic nerve injury, including direct trauma to the nerve, ischemic nerve damage, mechanical stretch or compression injuries, thermal damage from cement curing, compression from hematoma, and altered structural environment of the nerve after bony fixation. The underlying cause is unidentified in up to 50% of cases,^2^ highlighting the opportunity to better understand specific injury mechanisms as provided by this current report.

### Preoperative evaluation and anatomical considerations

Our case presents several unique and instructional pathoanatomic considerations. The through-and-through transection and retention of the cerclage wire within the nerve caused not only the disabling primary traumatic laceration but moreover ongoing compression and traction injuries to both the posterior tibial and the peroneal branches of the sciatic nerve, worsening her split nerve injury. The objective of surgical neuroplasty is to preserve the nerve in continuity and reduce the risk of further neurological deficits. Repairs with nerve grafting have the best outcomes within the first 6 months,^14,15^, and recovery following sciatic nerve repair at greater than 10 months postinjury is rare.^16^ Neuropathic pain symptoms have a higher likelihood of improvement even with delayed decompression. The injury location in our case was at a proximal segment of the nerve, which precluded mobilization of the nerve and end-to-end interposition. Nonetheless, we show the results of delayed nerve repair 1 year following injury using processed human nerve allograft. Given the multitude of repair options (e.g., neurolysis, graft repair, suture repair) the informed consent discussion should not be taken lightly.^17^ When feasible, suture repair has demonstrated higher rates of favorable (e.g., Grade III or better) functional recovery than graft repair.^18^ However, due to this patient’s Sunderland Type 4 injury with a relatively long end-to-end gap (Fig. 3), graft repair was necessary, making it crucial that we had determined her preference for allograft over autograft repair.

We showed the relevance of preoperative structural MRI/MRN for delineating the course of the sciatic nerve given its anatomical variations relative to the piriformis muscle at the hip level, and at its bifurcation site into the posterior tibial and common peroneal branches at the lower thigh or popliteal fossa level.^19,20^ MRN suppresses signals from vascular and adipose tissue to optimize selectivity for neural tissue properties and provides valuable information on the extent of neurodegeneration or regeneration. MRI/MRN have become part of the standard diagnostic algorithm for surgical neuroplasty and can even be considered prior to THA to better understand the surgical anatomy. In a 2014 case series by Wolf et al., MRN identified mechanical compromise and informed early surgical intervention and recovery in several patients, one with sciatic nerve constriction by cerclage wiring.^21^ In our case, CT reconstruction of the prior THA site also assisted in the visualization of the surgical anatomy of the bone, hardware, and cerclage wire.

In cases of complex sciatic nerve anatomy, neurosurgical consultation to assist with operative planning prior to THA or cerclage wire placement could reduce the risk of inadvertent neural injury. During THA, it is important to place cerclages wires with appropriate anatomical curvatures to the subtrochanteric area under direct visualization and create sufficient space between the femur and surrounding soft tissue prior to introducing the wire passer. The wire can be passed from the posterolateral direction, curving around the proximal femur anteromedially, to avoid the sciatic nerve located posteromedially.^10^

As shown in our report, whether the nerve is transected or compressed by the cerclage wire may not be fully appreciable on preoperative imaging. Imaging with reduced susceptibility to metal artifacts, combined with nerve tractography, could improve diagnostic accuracy and constitute important directions for near-term development. However, it is important for clinicians to have a high level of suspicion even in the absence of positive imaging findings.

### Intraoperative techniques and assessments

In the case of through-and-through nerve transection, maintaining control of the cerclage wire segments on both sides of the planned disconnection site is critical to preserving the safety of surrounding structures from inadvertent recoil when the wire is severed. Attention should be directed to protecting proximate neurovascular structures when dissecting and freeing the nerve from the penetrating wire. Due to the inherent rigidity of the wire and the inability for circumferential visualization, applying force or tugging maneuvers is discouraged to prevent serious occult injuries. Early comanagement with orthopedic surgery is recommended to evaluate safety and surgical approach. Our patient was more than 6 months removed from THA and did not have fractures or malalignment on preoperative CT, which permitted the removal of the wire without jeopardizing the stability of the arthroplasty. Had the sciatic nerve injury been identified earlier, coordination with orthopedic surgeons would have been essential to facilitate wire replacement prior to nerve decompression and repair.

Utilizing IONM modalities during THA should be considered in cases with complex anatomy. While the association between IONM and clinical outcomes after THA requires further validation, studies suggest that multimodal IONM during primary or revision THA may enable earlier identification of nerve injuries and real-time corrective strategies, thus decreasing the risks of permanent neuropathy.^22^

## Conclusions

We present a rare and unusual case of a penetrating transection of the sciatic nerve caused by femoral cerclage wire from prior THA associated with progressive sensorimotor deficits and neuropathic pain secondary to ongoing microtrauma, neural compression, and traction. Surgical pathoanatomy can be complex and underscoring the importance of careful preoperative planning in both primary THA and revision neuroplasty to optimize outcomes. Strategies include preoperative MRN to determine the anatomical course and characteristics of the affected nerve, early comanagement by neurosurgery and orthopedic surgery specialists, and multimodal IONM to reduce risks of neural injury and preserve function.

## Data Availability

Supporting data for the case report can be made available upon request to the corresponding author.

## Abbreviations Used

CT: computed tomography
EMG: electromyogram
IONM: intraoperative neurophysiology monitoring
M: motor scale
MRC: Medical Research Council
MRI: magnetic resonance imaging
MRN: magnetic resonance neurogram
MUAP: motor unit action potential
S: sensory scale
THA: total hip arthroplasty
USA: United States of America
